# Between roost contact is essential for maintenance of European bat lyssavirus type-2 in *Myotis daubentonii* bat reservoir: ‘The Swarming Hypothesis’

**DOI:** 10.1038/s41598-020-58521-6

**Published:** 2020-02-03

**Authors:** Daniel L. Horton, Andrew C. Breed, Mark E. Arnold, Graham C. Smith, James N. Aegerter, Lorraine M. McElhinney, Nick Johnson, Ashley C. Banyard, Robert Raynor, Iain Mackie, Matthew J. Denwood, Dominic J. Mellor, Sue Swift, Paul A. Racey, Anthony R. Fooks

**Affiliations:** 1 Animal and Plant Health Agency (Weybridge), Surrey, KT15 3NB United Kingdom; 2 School of Veterinary Medicine, University of Surrey, Daphne Jackson Road, Guildford, Surrey GU2 7AL United Kingdom; 3 School of Veterinary Science, University of Queensland, Brisbane, Australia; 4 Epidemiology and One Health Section, Department of Agriculture, Water and Environment, Canberra, Australia; 5 National Wildlife Management Centre, Animal and Plant Health Agency, York, YO41 1LZ United Kingdom; 6 Institute of Infection and Global Health, University of Liverpool, Liverpool, United Kingdom; 7 Scottish Natural Heritage, Great Gen House, Leachkin Road, Inverness, IV3 8NW United Kingdom; 8 Institute of Biological and Environmental Sciences, University of Aberdeen, Aberdeen, AB24 2TZ United Kingdom; 9 School of Veterinary Medicine, University of Glasgow, 464 Bearsden Road, Glasgow, G61 1QH United Kingdom; 10 Health Protection Scotland, Meridian Court, 5 Cadogan Street, Glasgow, G2 6QE United Kingdom

**Keywords:** Ecological epidemiology, Viral epidemiology

## Abstract

Many high-consequence human and animal pathogens persist in wildlife reservoirs. An understanding of the dynamics of these pathogens in their reservoir hosts is crucial to inform the risk of spill-over events, yet our understanding of these dynamics is frequently insufficient. Viral persistence in a wild bat population was investigated by combining empirical data and in-silico analyses to test hypotheses on mechanisms for viral persistence. A fatal zoonotic virus, European Bat lyssavirus type 2 (EBLV-2), in Daubenton’s bats (*Myotis daubentonii*) was used as a model system. A total of 1839 *M*. *daubentonii* were sampled for evidence of virus exposure and excretion during a prospective nine year serial cross-sectional survey. Multivariable statistical models demonstrated age-related differences in seroprevalence, with significant variation in seropositivity over time and among roosts. An Approximate Bayesian Computation approach was used to model the infection dynamics incorporating the known host ecology. The results demonstrate that EBLV-2 is endemic in the study population, and suggest that mixing between roosts during seasonal swarming events is necessary to maintain EBLV-2 in the population. These findings contribute to understanding how bat viruses can persist despite low prevalence of infection, and why infection is constrained to certain bat species in multispecies roosts and ecosystems.

## Introduction

Bats have been implicated as reservoirs for multiple viral, bacterial and protozoal pathogens of human and animal health importance, including Ebola virus, *Bartonella spp*., and rabies virus^1,2^. Aspects of the ecology and demography of bats such as forming high-density aggregations, and flight permitting long-distance movement, may play a role in this ability to maintain a variety of pathogens^2^. Mechanisms of pathogen persistence at the population level are often poorly understood, particularly when infection elicits a long lasting protective immune response^3^.

Rabies virus is one of a growing number of recognised lyssavirus species that are all capable of causing fatal encephalitis^4^. The majority of these lyssaviruses are associated with bats^5^. Although the full public and animal health impact caused by infection with lyssavirus from bats worldwide is unknown, they are an increasing cause for concern. In the Americas, rabies virus occurs in bats, and bat-derived rabies has become the leading cause of human rabies in the United States^6,7^. On every other continent where bats occur at least one distinct lyssavirus has been detected in bat populations, and the antigenic differences between some of the more divergent lyssaviruses from vaccine strains, mean there is the potential for reduced vaccine efficacy^8,9^ against those strains. Understanding infection dynamics in the reservoir host is critical to understanding and managing the public and animal health risks, and yet fundamental questions remain concerning the dynamics and maintenance of lyssaviruses in bat host species. Critically, the low frequency of detected virus excretion and relatively short infectious periods suggest that the mechanism of maintenance of infection within a reservoir population is not resolved and has led to suggestions that the viral infection dynamics in bats may differ fundamentally from those in terrestrial carnivores.

Rabies virus (RABV) in bats has been well studied compared to other lyssaviruses and other bat pathogens. In the US, over 1500 RABV-infected dead bats were reported each year between 2008 and 2012 through passive surveillance, from nearly all of the 39 commonly encountered North American species^6,10^. In addition, surveillance activities have shown a low but consistent seroprevalence for antibodies to rabies. A study of longitudinal targeted sampling data of rabies in big brown bats (*Eptesicus fuscus*) suggested that maintenance is dependent on host ecology, and in particular, on hibernation^11^. A slower metabolic rate in the host and slower virus replication during hibernation prevents early death of the host and epidemic fade-out of the virus. This implies that hibernation is essential for rabies virus maintenance. The hibernation theory is consistent with *in-vitro* and *in-vivo* data that show lower virus replication at lower temperatures in North American bat species^12,13^. However, it is not clear whether this hypothesis applies to lyssaviruses in other bat species in other regions with different host ecology and climates.

Of the European bat lyssaviruses, type 1 (EBLV-1) has been the most studied but has been detected at a much lower frequency than RABV in the Americas, with a total of over 1000 cases reported over 30 years primarily in *Eptesicus* spp^14^. Studies of EBLV-1 dynamics have demonstrated seasonal and inter-annual variation in seroprevalence of between 11.1 and 40.2%, and demonstrated roost size and species richness are associated with higher seroprevalence^15–17^. Metapopulation models have suggested that inter-species transmission and migration behaviour have contributed to persistence of that virus^18,19^.

Unlike EBLV-1, EBLV-2 has been detected exclusively in two species, Daubenton’s bat (*Myotis daubentonii*) in the UK and the Pond bat (*Myotis dascyneme*) in the Netherlands. Fifteen cases of European bat lyssavirus type 2 (EBLV-2) have been detected in Daubenton’s bats (*Myotis daubentonii*) in the UK, the first in 1996 and the most recent in 2017. Sero-surveillance has demonstrated a seroprevalence in the UK of up to 4%^14,20–22^. Daubenton’s bats are widespread temperate insectivorous bats with a range from Europe to Japan^23^. They are ubiquitous in Northern Europe, with analysis of genetic diversity suggesting panmixia across its range in continental Europe^24^. During the summer many communities demonstrate a degree of sexual segregation with adult females forming maternity roosts and males dominating in ‘bachelor’ roosts. Whilst movement between roosts is common in spring and summer, this usually appears to be restricted to resorting of individuals across a local network of sites within relatively well defined summer communities^25^. A notable feature of the lifecycle of this species involves mixing in large numbers in the autumn^26–28^. This autumnal ‘swarming’ behaviour may represent lekking behaviour and is considered important for mating, as well as often being associated with hibernacula used by cave hibernators such as Daubenton’s bats^29,30^. The aggregation of bats during the summer natal period, and autumnal swarming present suitable conditions for direct disease transmission with increased contact between individuals.

The death of bat-workers due to EBLV-2 infection after being bitten by Daubenton’s bats *(M*. *daubentonii)* in the UK and Finland^31,32^ demonstrate the zoonotic potential of EBLV-2 and focus attention on managing the public and animal health risks. In the absence of detailed disease prevalence data, seroprevalence determined by sampling live bats has been used as a surrogate for EBLV-2 persistence in bat populations^33^. This assumes that it is possible for a bat to be exposed to the virus and seroconvert to a level detectable by virus neutralising antibody tests, and that the tests are specific for the virus being studied^34^. Based on our understanding of lyssavirus transmission in all mammals, infection usually leads to fatal encephalitis. This clearly occurs in naturally and experimentally exposed bats^35,36^. However, numerous serology studies have shown lyssavirus antibodies in healthy bats leading to the conclusion that they have either successfully controlled infection or been exposed to sufficient virus to seroconvert without active infection in the bat’s nervous system. Proposed mechanisms in the latter case include aerosol exposure to virus in the roost^37,38^ or repeated exposure to virus during normal roost behaviour such as allogrooming. A similar phenomenon has been reported in the Peruvian Amazon, where antibodies detected in humans were attributed to nonfatal exposure to rabies virus from vampire bats^39^. In the absence of conclusive observational or experimental data regarding mechanisms of persistence in a population, it is necessary to develop and test hypotheses for EBLV-2 transmission and seroconversion through infection dynamics modelling^3^.

Seroprevalence data for EBLV-2 in Daubenton’s bats was collected from over 20 roost sites in England and Scotland over nine years and multivariable statistical models and Bayesian disease dynamics models were fitted to these data to test hypotheses of viral persistence. The results provide a new explanation for maintenance of viral infections in this species with implications for understanding persistence of other infectious pathogens in bat populations, and directing health policies for lyssaviruses.

## Methods

All methods were carried out under licence in accordance with relevant guidelines and regulations from the appropriate competent authorities (UK Home Office, English Nature and Scottish Natural Heritage) and all experimental protocols were approved by the Animal and Plant Health Agency’s ethics committee.

### Data collection and statistical models

Serological and ecological data from Daubenton’s bats used to inform and test the disease dynamics model were collected as part of a prospective nine-year, serial cross-sectional, mark-recapture surveillance study across sites in England and Scotland. The whole study system included a total of twenty roosts over a wide geographic area of Northern England and Scotland. These roosts varied in size and included those in man-made and natural structures. To determine factors associated with seropositivity (using statistical models), data from all roosts was included. For the models of disease dynamics, in order to represent a set of bat roosts with the potential for between-roost mixing, four roosts in the North West of England were included in the model. These were chosen due to reliable size and access for annual sampling, to allow the population size and number infected to be estimated from annual sampling (Roosts A to D, Table 1 and S1). The selected roosts were far enough apart such that they were not part of the same summer roost networks i.e. individuals from the selected roosts were most likely to mix with others from unidentified roost sites. Average approximate straight line distance between these four roosts A-D was 42 km (range 10–70 km). Previous studies have demonstrated a mean roost home range of 0.2 km^2^ for female *M*. *daubentonii*^25^ and no bats were captured at more than one roost of the four selected roosts A-D during the study. Sampling was restricted to May-September due to access to bat populations, and to avoid disturbance during hibernation. Parameters describing roost population size and mortality rates for adults and juveniles at each roost (Table S1) were derived empirically using capture mark-recapture methods (detailed in Supplementary Information) whilst host reproductive rates and incubation periods were taken from the literature (Table 2).

**Table 1:** Bats sampled and test results from roosts used for models of disease dynamics.

Roost	Number of sampled individuals	Number of seropositive individuals	Percent positive
A	202	12	5.9%
B	54	4	7.4%
C	51	6	11.8%
D	135	21	15.6%

**Table 2:** Input parameters for the model.

Parameter	Value	Source
Transmission rate	0.0058 per infected per day	Fitted to data
Proportion exposed that become infectious	0.15 (i.e. 0.85 of infected bats move to resistant state)	George *et al*.^11^
Disease-induced mortality rate	1/6 (i.e. mean 6 days before death)	Freuling *et al*.^49^, Johnson *et al*.^35^
Incubation rate	1/24 (main transmission i.e. 24 day incubation period) 1/48 during hibernation phase	Freuling *et al*.^49^, Johnson *et al*.^35^, George *et al*.
Immunity rate	1/14 (main)	Freuling *et al*.^49^, Johnson *et al*.^35^
Host reproductive rate	1 per year	Dietz *et al*. 2009

Samples from a total of 1839 *M*. *daubentonii* bats were tested for evidence of anti-EBLV-2 neutralising antibody and 1680 for virus excretion. Antibody titres were assessed using a modified fluorescent antibody virus neutralisation test (FAVN) in BSL3/SAPO4 containment. A neutralisation titre of 1:15 was used as a threshold for seropositivity (see Supplementary Information). The antibody titre for the first sample from each bat was used for the models, and the repeated sampling was used to establish the longevity of the antibody response and to detect evidence for seroconversion during the study.

The presence of virus in oropharyngeal swabs was assessed using both real-time RT-PCR targeting the nucleoprotein gene and standardised tissue culture tests on neuroblastoma cells, in BSL3/SAPO4 containment (See Supplementary Information for details).

Antibody titres were reported as endpoint reciprocal titres, and analysed using univariate and multivariable regression models in STATA and SPSS. Univariate regression models were used to assess the effect of individual traits (weight, age, sex, reproductive status and time) on the odds of being seropositive, and then multivariable logistic regression models were used to try to distinguish the relative effect of each of those multiple traits. Several regression models were applied, including combinations of the following terms: weight, age, sex, reproductive status, month and year (Table S4).To account for possible differences between countries, each univariate model had a random effect for country included (intercept, or intercept plus slope for continuous variables weight and forearm). For assessing roost differences, univariate models were fitted to the roosts in each country (England/Scotland) separately. Model fit was assessed using the Akaike information criterion, Hosmer Lemeshow test and c-statistics.

### Models of disease dynamics

The modelling of within-roost transmission was designed to avoid unnecessary complexity while fitting to the observed data. A standard SEIR model was followed with susceptible, exposed, infectious and resistant states^40^. Bats that are exposed enter a latent period from which they either; become infectious and die shortly afterwards, or recover and become seropositive for lyssavirus (resistant). The model assumes that bats cannot have productive (infectious) rabies and then recover. This is in line with extensive evidence from other species, and experimental evidence from bats, and with previous modelling studies^11^. A multi-roost epidemiological model with ordinary differential equations was used (see Supplementary Information for details). The model included three separate seasonal transmission phases: firstly, a main transmission period (May–Aug), during which bats are active and within roost transmission occurs; this is followed by an autumnal period that included swarming (Sept–Nov), during which bats mix with individuals from other roosts, and between roost transmission of infection may occur; thirdly, there is a hibernation period (Dec–April), during which there is assumed to be no transmission. Input parameters included host reproductive rate, a proportion exposed that become infectious, an incubation period, a rate for development of immunity and a disease-induced mortality rate (Table 2). There were two age classes of bat in the model, juvenile and adult, so that differences in mortality and reproduction rates between juveniles and adults could be represented. Both natural and density-dependent mortality were represented in the model, with survival rates at each roost and population size estimated from data collected in the study. Density-dependent mortality was set such that population sizes remained approximately constant throughout the simulations, matching those from mark-recapture studies.

An additional parameter was included to represent the degree of between roost transmission events, with values between 0 (no between roost transmission) and 1 (rate equal to within-roost transmission). In order to estimate the transmission rate, an approximate Bayesian computation (ABC) approach^41^ was used to find the values of the transmission rate that produced the best fit to the observed serology data (the error term in the ABC metric was given by the sum of (observed-expected)^2^/expected for each year for each roost, where expected was given by the model output). In addition, the model was used to determine statistical evidence of between-roost transmission. This was undertaken by implementing two models in the ABC framework, one including between-roost transmission (to simulate mixing during autumn i.e. swarming) and one with no between-roost transmission. A *model-choice* parameter was also included in the ABC algorithm to reflect the proportion of model outputs that were sampled from the swarming model (vs the non-swarming model). The final output of the ABC framework was a probability distribution of (i) the between bat transmission rate and (ii) the likelihood that the model with between roost transmission was a better fit to the data than the one without.

Although there is a lack of support for density-dependence in bat rabies^42^, previous studies have not provided consensus on the use of density-dependent or frequency-dependent models. Therefore, a frequency dependent model was also fitted to the data to verify that the findings of the model in terms of swarming were independent of the assumption of frequency or density-dependence on transmission (see Supplementary Information).

## Results

Following removal of samples with insufficient volume or quality, 1839 serum samples were suitable for testing for anti-EBLV-2 antibodies, and 1680 oropharyngeal swabs for virus shedding using PCR and virus culture (see Supplementary Information). Corresponding morphometric, sex and age data were collected for each bat.

### Demographics

**Figure 1:**
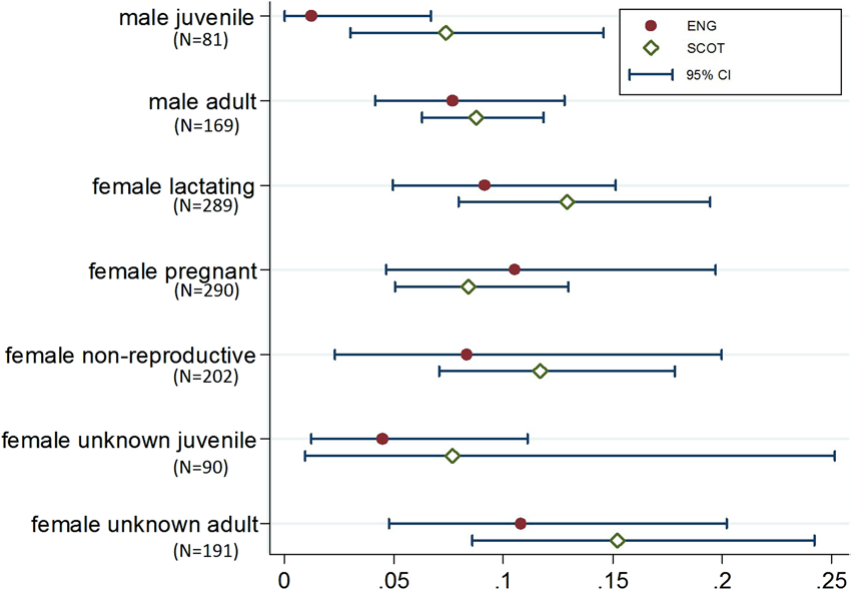
Seroprevalence by age and reproductive status (with 95% CI) for bats from England and Scotland.

The proportions seropositive by age, sex and reproductive status are illustrated in Fig. 1. Univariate and multivariable regression models were used to investigate which factors increase or decrease odds of a bat being seropositive to EBLV-2 (Table 3). Results of the best fit multivariable models are largely consistent, and those models included combinations of age, sex, reproductive status and year (Table 3). Models were selected where the Hosmer-Lemeshow test was not significant at the 5% level and c-statistic values were between 0.70 and 0.74 (Table S4). Plots of residual values versus predicted values are in Supplementary Information.

**Table 3:** Correlates of seropositivity against EBLV-2 in Daubenton’s bats.

Covariate	Positive/Total	% positive	Univariate		MODEL A		MODEL B		Model C	
			OR	p	OR (95% CI)	p	OR (95% CI)	p	OR (95% CI)	p
**Weight**										
	167/1839	9.1%	1.23 (1.07–1.42)	**0**.**01**						
**Age**										
Juvenile	14/292	4.8%	1		1		1		1	
Adult	153/1548	9.9%	2.18 (1.24–3.82)	**0**.**01**	2.06 (1.09–3.88)	0.03	2.02 (1.11–3.67)	**0**.**02**	2.03 (1.09–3.77)	**0**.**03**
**Sex**										
Female	108/1062	10.2%	1		1		1		1	
Male	59/778	7.6%	0.72 (0.51–1.00)	**0**.**05**	0.6 (0.39–1.12)	0.12	0.70 (0.49–1.01)	0.06	0.57 (0.34–0.96)	**0**.**04**
**Repro status**										
Unknown	87/1059	8.2%			1				1	
Female lactating	32/289	11.1%			0.99 (0.51–1.92)	0.97			0.71 (0.39–1.30)	0.23
Female pregnant	26/290	9.0%			0.82 (0.38–1.74)	0.60			0.54 (0.29–1.00)	**0**.**05**
Female non-reproductive	22/202	10.9%			0.81 (0.41–1.58)	0.54			0.74 (0.39–1.40)	0.36
**Month**										
June	50/432	11.6%			1.79 (0.97–3.32)	0.06				
July	56/537	10.4%			1.43 (0.87–2.34)	0.16				
August	49/675	7.3%			1					

Model A (AIC 1072) includes age, sex, reproductive status, month, year and random effect for location, Model B (AIC 1069) includes age, sex, year, random effect for location. Model C (AIC 1107) includes age, sex, reproductive status and random effect for location. The Hosmer-Lemeshow test is not significant at the 5% level for any of the models and c-statistic values are between 0.70 and 0.74 (Table S4).

In univariate models, both weight and age significantly increased the odds of a bat being seropositive. However, in the multivariable models, the effect of weight is not significant, and removing it does not alter the fit of the models. Age remains significant in all three best fitting models (OR 2.02 95% CI 1.11–3.67, p = 0.021) (Table 3). This age-related increase in seroprevalence is consistent with horizontal transmission within the population. In both univariate and multivariable models, males are less likely to be seropositive than females, but the effect is only marginally significant (OR 0.72 95% CI 0.51–1.00, p = 0.049).

### Temporal variation

Sampling was undertaken in May through to September, with 88% of sampling in the months of June, July and August. Parturition in Daubenton’s bats tends to occur in synchrony within a maternity roost, during a narrow time period (the ‘birth pulse’), usually in May or June (unpublished observations). To investigate potential effects of reproduction on seroprevalence, the effect of both month and female reproductive status (pregnant/lactating/non reproductive) on seroprevalence was investigated. No significant difference in total or female-only seroprevalence was detected between June (OR 1.79 95% CI 0.97–3.32, p = 0.06) and July (OR 1.43 95% CI 0.87–2.34, p = 0.16) when compared with August (Table 3, Model A). In multivariable models including sex and age, pregnant females were less likely to be seropositive (OR 0.54, p = 0.05).

**Figure 2:**
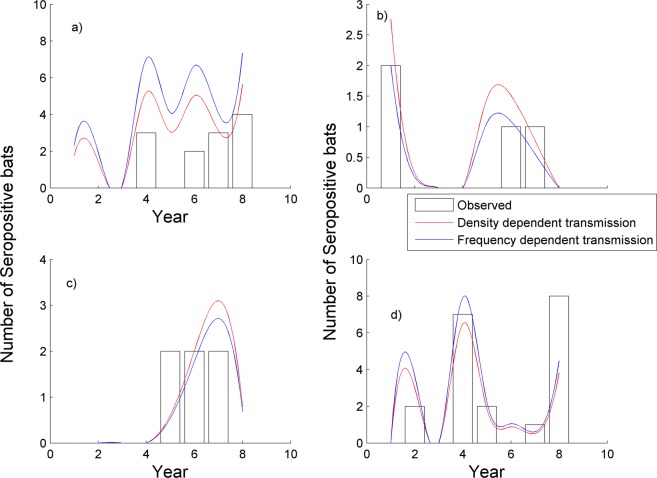
The fit of the model versus the observed data on the number of serological positive bats each year by roost, (**a**–**d**) (see Table 1).

There was evidence for inter-annual variation in total seroprevalence, ranging from 3.2% in 2009 to 17.7% in 2005 suggesting fluctuations in disease prevalence (OR 0.15 95% CI 0.08–0.30, p < 0.001) (Table S2). These fluctuations are also observed in the model outputs, and the observed data for individual roosts A-D (Fig. 2).

### Location

There was statistically significant variation in the number and proportion of seropositive bats caught in each roost but no overall spatial pattern or trend. For example, seven roosts had a higher proportion of positive bats when compared to the largest English roost A (n = 202 bats, 5.9% positive), with the highest being East 1 (n = 30, proportion positive 30% OR 3.55, p = 0.001) (Table S3). Small sample sizes from each site, and different sampling strategies used in Scotland and England preclude firm conclusions regarding geographical variation. Thus in multivariable analyses a random variable is included for location. See Supplementary Information Table S2.

### Antibody longevity

Ringing and re-capture allowed longitudinal serological sampling of some individual bats. A total of 176 bats were sampled more than once. The majority of bats (n = 150) were seronegative at all recapture events. 20 resampled bats were either seropositive at multiple recaptures, or seroconverted during the study, and only six had titres that dropped below the threshold at recapture. Eight seropositive bats were recaptured more than twice; six of those eight maintained high titres for three years, and four maintained high titres for over four years.

### Virus shedding

Of the oropharyngeal swabs from bats tested for lyssavirus genetic material during the course of the study (n = 1680), a single sample was positive by RT-PCR for EBLV-2 and none were positive by virus isolation.

### Disease dynamics models

To investigate the importance of autumnal swarming in maintenance of infection in the population, two models were included in the ABC framework, one including between-roost transmission after the summer season (to simulate mixing during autumn swarming) and one with no between-roost transmission.

The ABC procedure showed that the swarming model fitted the data better than the model without swarming, and was favoured in all model runs (giving a model choice parameter of 1.0). Without between-roost mixing in the autumn, there was rapid fade out of infection from the small roosts (B and C).

The fit of the model output to the observed data is shown in Fig. 2. (Fig. 2) The model tended to underestimate the prevalence in the smaller roosts (Fig. 2b,c) and slightly overestimate the prevalence in the larger roosts (particularly Fig. 2a).

For the density-dependent transmission model, the value of the transmission rate (beta) was very low, with an estimate of 0.0061 and a 95% credible interval (CrI) of 0.0060–0.0062. The deterministic set of differential equations produced estimates of the number of seropositive bats that were clearly very sensitive to small changes in the value of the transmission rate. The estimate for the between-roost transmission rate was equal to 0.85 (95% CrI: 0.79–0.96), suggesting a lower rate of between-roost transmission compared to within roost transmission (Figure S1).

For the frequency dependent transmission model, the ABC fitting also showed a better fit when swarming occurred, with the model choice parameter equal to 0.98 for the between-roost transmission model. For this model, the within roost transmission rate (1.10, 95% CI 1.00–1.17) was close to the between-roost transmission rate (0.97, 95% CI 0.57–1.62) (Figure S2).

## Discussion

The complex causal relationships between life history traits, spatial structure and infection dynamics of pathogens and their hosts are poorly understood^43^. This analysis of long-term seroprevalence data demonstrates long-term maintenance of a fatal zoonotic pathogen in a widespread bat population. Furthermore, this analysis suggests an explanation for maintenance that depends upon bat behaviour producing seasonal variation in the rate of contacts among bats, which results in changes to the scale of contact patterns. Finally, and relevant to the study of other host-pathogen systems, these results demonstrate that a holistic approach involving pathogen and host ecology are required to better understand viral disease dynamics in bats.

European bat lyssavirus type 2 has been repeatedly detected in Daubenton’s bats in the UK over the past 20 years. However, the low prevalence and dispersed location of different isolations gives no indication of how the virus persists in the bat population. The evidence presented here, of seroconversion and antibody persistence in healthy bats, combined with higher odds of adult bats being seropositive, indicates that EBLV-2 is likely to be endemic at a low level within British *M*. *daubentonii*, with horizontal transmission between individuals. This maintenance of infection in the absence of significant observed disease or mortality needed to be investigated to fully understand the public health implications. A proportion of the human population and domestic animals come into contact with bats, and the risk posed by healthy bats is unknown^44^. The traditional models of rabies pathogenesis include a variable incubation period, followed by a single short symptomatic infectious period, and death^45^. This could also be the case for bats based on experimental observations^35,46^. Despite lack of evidence, persistent or intermittent viral shedding in healthy bats has been previously proposed as a possible mechanism for maintenance. The very low proportion of oral swabs from bats in this study positive for EBLV-2 by sensitive molecular assays and culture, concurs with other studies suggesting persistent infection is unlikely^47^.

Previous studies, both experimental and in-silico, have suggested hibernation as a potential mechanism for virus maintenance in temperate bat species^11–13,48^. Lower host metabolic rate slows virus replication, extending incubation periods and therefore allowing virus persistence between transmission periods. Including hibernation in the present study, however, failed to prevent epidemic fade out. Slower viral replication caused by reduced metabolic rate may well cause delayed incubation periods in this EBLV-2 system, but these data suggest this alone is not sufficient to maintain infection with effective population sizes equivalent to the observed roost sizes. Indeed, it is pertinent to note that seroprevalences at observed roosts were estimated to be zero on multiple occasions. An additional consideration in heterotherms is the apparent conflict between energy conservation and production of an immune response during torpor or hibernation^49^. The rise of White Nose Syndrome, a fungal infection caused by *Pseudogymnoascus destructans*, has focussed attention on this issue, as the infection and production of a protective immune response may actually disturb torpor, with associated detrimental effects^50^. It is possible that the long-lived antibody seen here in Daubenton’s bats is also related to differences in the immune response of heterotherms. Another characteristic of bats is flight, which has also been hypothesised to influence host-pathogen interaction through elevated temperature^51^, and these differences are worthy of further investigation.

The frequency dependent model also suggests that there could be an effective population size threshold under which infection would be expected to fade out, and both frequency and density-dependent models found mixing between communities is essential in order for the models to provide the best match to the observed data. Autumnal swarming presents an ideal opportunity for this, as it is characterised as it is by intense chasing flights, presumed social contact between all bats, and direct contact during mating. It has been described for at least 26 bat species from seven genera in Europe and North America^30^ in and around sites often also used as hibernacula and is frequently observed in all common British cave hibernating *Myotis* and *Plecotus* spp. Late summer to early autumn is also the peak time period when diseased and moribund bats infected with EBLV-2 are reported in the UK^21^. Swarming sites are thought to be ‘hotspots’ for gene flow between otherwise isolated bat populations^52^ and may operate from regional to international scales, potentially allowing transmission and maintenance of infectious disease. Indeed, van Schaik and Kerth^53^ have shown the relevance of inter-roost contact during swarming for transmission of *Spinturnix* mites in three European *Myotis* species including *M*. *daubentonii*. Albeit in a different ecological context, the importance of inter-roost mixing has also been demonstrated for the maintenance of rabies virus infection in populations of haematophagous bats^54^.

Relevant contact patterns and hence effective population size for maintenance of infection may vary among different pathogens for the same host species depending on the mode of transmission, infectious period and other epidemiological parameters. A behavioural study of *M*. *daubentonii* in England showed males to have greater intergroup contact than females during summer and predicted higher exposure of males than females to infectious disease^25^. However, the observations in the present study suggest a slightly higher seroprevalence in females than males and suggests that summer intergroup contact by males, as suggested by August *et al*.^25^, is not a significant driver for EBLV-2 dynamics in *M*. *daubentonii* in Britain, although it may still be relevant for other pathogens. In contrast, male dispersal behaviour has been suggested as a driver of rabies virus transmission in vampire bats (*Desmodus rotundus*) in South America^55^. More consistent with the data observed here, is a more equal ratio of male-female contacts resulting in disease transmission, as could occur during swarming.

There is no consensus on whether transmission of a pathogen such as EBLV-2 among bats in a system such as the one studied here would be predominantly density- or frequency-dependent. This question has been explored previously in other systems, for example in haematophagous bats where culling is used as an attempted control method^56^. However the model structure used here requires an assumption to be made on the predominance of one or other scenarios. Therefore both scenarios were modelled and, while no substantial difference in model outputs was observed for the final fitted models, they do behave differently for changes in roost size. For the density-dependent models, the basic reproduction rate (R0) of pathogens will depend linearly on the population size. In the present study, simulations suggest that lyssavirus infection could not be sustained for roost sizes less than around 200 without between roost transmission, given the fitted estimate of the transmission rate for density-dependent transmission. For frequency dependence, there was little change in R0 for changes in roost size. In this case, there was no threshold population size for maintenance of infection as such, but the inclusion of swarming enabled the model to provide a better fit to the data. There was not sufficient power in the data from the present study to infer whether density- or frequency-dependence was more likely. Spatially explicit models as proposed by Begon *et al*.^57^, and incorporating the effect of sex on seroprevalence into models may prove informative in the future.

Managing the public health and conservation impacts of wildlife diseases such as EBLV2 remain a challenge. Ongoing habitat fragmentation is predicted to affect large-scale behavioural patterns such as swarming in *M*. *daubentonii*^27^. The potential effect of changes such as this on infection dynamics and host population viability are highly uncertain and hence mathematical models that incorporate infection and behavioural dynamics as presented here, may be crucial to the successful management of public health risk as well as conservation.

## Supplementary information

Supplementary Information.

Supplementary Dataset 1.

## Data Availability

The data that support the findings of this study are disclosed in the paper and its Supplementary Information Files.
