# Supplementary material for: Between roost contact is essential for maintenance of European bat lyssavirus type-2 in Myotis daubentonii bat reservoir: ‘The Swarming Hypothesis’
Source: Sci Rep. 2020 Feb 3;10:1740. doi: 10.1038/s41598-020-58521-6 (PMC6997190; doi:10.1038/s41598-020-58521-6)
Supplement: Supplementary file 1 — Supplementary Information. [file 41598_2020_58521_MOESM1_ESM.docx]

# Supplementary Information

# Title

**‘Between roost contact is essential for maintenance of European bat lyssavirus type-2 in *Myotis daubentonii* bat reservoir: ‘The Swarming Hypothesis’**

# Authors

DL Horton^1,2#^, AC Breed^1#*^, ME Arnold^1^, GC Smith^3^, JN Aegerter^3^, LM McElhinney^1,4^, N Johnson^1,2^, AC Banyard^1^, R Raynor^5^, I Mackie^6^, MJ Denwood^7^, DJ Mellor^7,8^, S Swift^6^, PA Racey^6^ and AR Fooks^1,4^

# Methods

Bat capture and sampling was undertaken under licence from the appropriate competent authorities (UK Home Office, English Nature and Scottish Natural Heritage) and after independent ethical review.

## Bat capture and sampling

Bats were caught using approved methods from free flight and at their roosts [1]. Catching from free flight usually occurred in rivers, where this species can commonly be found commuting and foraging close to the surface of the water. Unfortunately, this is labour intensive work and can be subject to low catching success. In addition, this species is known to commute some distance to forage, and samples associated with capture from free flight are difficult to confidently associate with a spatially explicit ‘home’ community. Thus the majority of work was undertaken at summer roosts; the only locations holding worthwhile densities of bats suitable for sampling. As the majority of known Daubenton’s roosts are maternity sites, dominated by adult females rearing young, samples from adults were female biased. Captured bats were described, noting; sex, age (adult/juvenile; noting the ossification of the phalangeal joints), reproductive condition (pregnant or lactating; by palpation of the abdomen), forearm length (0.1mm) weight (to 0.1g) and any existing ring marking for mark-recapture models (see below). Unmarked bats were marked with a unique ring (2.9mm Alloy; Mammal Society/BCT, England) followed by two oropharyngeal swabs, one for virus culture (in virus transport medium) and one for RT-PCR (in RNA Later), and a blood sample was taken from either the brachial vein or uropatagial vein (see Racey et al. 2011[2] for further details). Preserving the integrity of samples was achieved by ensuring samples were maintained at 4-10 °C using temperature controlled packaging. Bats were released at the point of capture once they had fully recovered from the procedure. In both England and Scotland, previously sampled bats were prioritised to ensure they were sampled when caught. Site selection protocols differed between England and Scotland.

In England, bats were caught from over 20 sites across six counties from 2003 until 2012 (excepting 2007). A mean of 125 bats were caught each summer over a three month period (see [3] for further details). The sampling approach combined three aspects; sites known a-priori to host disease (i.e. identified through a passive surveillance scheme), two sites sampled in most years to establish longitudinal data on disease prevalence, and additional sites selected at random and sampled or resampled to ensure some geographical structure. Site selection was constrained by the number of known roost locations and changing access permissions, as well as the inevitable spontaneous abandonment of known roosts typical for this species.

In Scotland, a mean of 240 bats were caught per year from 2005 to 2010 inclusive using site selection made within a spatially stratified framework extending across the full range of the species in Scotland. The antibody titre for the first sample from each bat was used for the statistical models, and the repeated sampling was used to establish the longevity of the antibody response and to detect evidence for seroconversion during the study.

### Detection of antibodies to European bat lyssavirus type 2

Blood was allowed to clot, the serum was then separated by centrifugation and then refrigerated before for the detection of antibodies to European bat lyssavirus Type 2 (RV1332) using a modified fluorescent antibody virus neutralisation test (mFAVN) in BSL3/SAP04 containment. A fixed quantity of virus was mixed with serial dilutions of serum before inoculation in cell culture. The endpoint dilution where neutralisation ceases was detected using a pan-lyssavirus fluorescent antibody, and reported as a reciprocal titre. There are no internationally agreed cut-offs for interpreting EBLV antibody results, so a threshold (reciprocal titre of 1:15) was chosen by comparison with the standard rabies antibody serum (0.5IU/ml) which routinely has a similar titre when tested against homologous standardised rabies virus (CVS). Cross reactivity between other lyssaviruses is theoretically possible at low levels [Horton 2010] but no other lyssaviruses have been reported in the UK despite consistent surveillance effort [wise 2017].

### Oropharyngeal swab analysis

Two oropharyngeal swabs were taken from each bat. One was placed in RNA preservative (RNALater, Qiagen) and tested by sensitive quantitative real time RT-PCR (for viral RNA) targeting a conserved region of the nucleoprotein gene (primers below) following RNA extraction using a column based kit (HiPure, Roche) [4] and one was placed in virus transport medium, and tested by the OIE prescribed tissue culture inoculation test (for the presence of live virus) on neuroblastoma (N2A) cells in BSL3/SAP04 containment [5]. Host gene controls and virus positive controls were used in the molecular assays as described previously [Harris 2009]

## Demographic analyses

Demographic rates were estimated from the recapture data (using unique ring markings) for four sites in England (A, B, C and D). A Multistate model was used in in the program MARK to derive annual estimates for both age (adult and juvenile) as well as sex based demographic classes. [6]. Data from sampling events conducted within the same year were pooled as they were often only separated by a few days. At each roost the top models (i.e. AICc < 2) were selected and used to produce model-averaged estimates of survivorship for adult and juvenile age classes and density dependent mortality (table S1). Estimates of male and female survival differed among roosts, though evidence for this was often weak, which may be a consequence of most roosts being associated with nursery sites and a lower likelihood of recapturing males. The epidemiological modelling did not require sex-specific survivorship parameters for each age class and the global age-based survivorship rates for each roost were used as the key estimate (Table S1). Most roost sites (A, B, D) also had sufficient data to describe strong differences between age classes, and reliable estimates of annual rates of survival for both age classes could be produced for two roosts (A, B). Unfortunately other roosts lacked sufficient data to ensure reliable annual estimates and fixed survivorship, so rates for each roost were estimated as model weighted averages.

The top performing models were also used to specify informative POPAN models estimating the size of each population (adults and juveniles), assuming a closed population. These lacked any strong trend in annual estimates (except for roost A) and were summarised into simple static descriptions for the abundance of bats at each site; allowing us to identify and contrast the epidemiological dynamics at large and small roosts. At roost A, our most sampled site, our assumption of a closed population may be inappropriate, though we have also estimated its population size using the same approach as the variation in estimates ranged from 150 to 250 individuals.

Table S1

| Roost | Survival estimate (SE) | | | Number of captured individuals | Number of capture occasions | Abundance estimate | Estimate of density dependent mortality |
| --- | --- | --- | --- | --- | --- | --- | --- |
|  | Adult | Juvenile | Overall |  |  |  |  |
| A | 0.77 (0.03) | 0.69 (0.09) | 0.76 (0.03) | 252 | 7 | 200 | 6.5 x 10^-6^ |
| B | 0.79 (0.05) | 0.64 (0.13) | 0.77 (0.05) | 96 | 7 | 50 | 2.4 x 10^-5^ |
| C | n.d. | n.d. | 0.76 (0.19) | 61 | 4 | 60 | 1.8 x 10^-5^ |
| D | 0.72 (0.03) | 0.52 (0.14) | 0.71 (0.03) | 169 | 6 | 150 | 8.3 x 10^-6^ |

n.d. = juvenile and adult rates not calculated due to low number of captured occasions

## Mathematical models of disease dynamics

### Model parameterization

In order to represent a set of bat roosts with the potential for between-roost mixing in autumn, four roosts were included in the model, for which the population size and number infected could be estimated from annual sampling. These sites were chosen as they were within the expected distance for contact via autumnal swarming but not during spring-summer. Movements of marked bats between sampled roosts were only detected on two occasions from a regional pool of 1345 marked bats. Population size each year for each roost was estimated from capture mark-recapture studies, and the average population size over the time period monitored was used in the model (Table S1)

In order to estimate the transmission rate, an approximate Bayesian computation (ABC) approach was used to find the values of the transmission rate that produced the best fit to the observed serology data. In addition, the model was used to determine whether there was statistical evidence of between roost transmission. This was undertaken by implementing two models in the ABC framework, one including between-roost transmission (to simulate mixing during swarming) in and one with no between roost transmission. A “model choice” parameter was also included in the ABC algorithm to reflect the proportion of model particles were sampled from the swarming model (vs the non-swarming model). The final output of the ABC framework was a probability distribution of (i) the between bat transmission rate and (ii) the likelihood that the model with between roost transmission was a better fit to the data than the one without (Figures S1 and S2).

**Model equations**

1. Main transmission season (1 May – 31 August)

This follows a standard SEIR model, with a Susceptible (S), exposed (E_I_ and E_R_), resistant (R) and infectious (I) states. Bats are divided into 2 age groups: juvenile (up to 1 year old) and adult females (>1 year old), with age group denoted by subscript *j (j=1,2* for juvenile/adult respectively*).* Roost is denoted by subscript *k*.

$$\frac{dS_{jk}}{dt}=-\beta S_{jk}I_{jk}-\mu_{j}S_{jk}-\varphi_{k}N_{k}S_{jk}$$

$$\frac{dE_{Rjk}}{dt}=\left( 1-\rho\right)\beta S_{jk}I_{jk}-\left( \sigma_{R}+\mu_{j}+\varphi_{k}N_{k} \right)E_{Rjk}$$

$$\frac{dE_{Ij}}{dt}=\rho\beta S_{jk}I_{jk}-\left( \sigma_{R}+\mu_{j}+\varphi_{k}N_{k} \right)E_{Ijk}$$

$$\frac{dR_{jk}}{dt}=\sigma_{R}E_{Rjk}-\mu_{j}R_{jk}-\varphi_{k}N_{k}R_{jk}$$

$$\frac{dI_{jk}}{dt}=\sigma_{I}E_{Ijk}-\nu I_{jk}-\varphi_{k}N_{k}I_{jk}$$

There is a single birth pulse at the beginning of the main transmission season, at which point the juvenile bats from the previous year progress to the adult age class. Delaying the date of the birth pulse made negligible difference to the behaviour of the model

1. Swarming season (1 September – 30 November)

This model follows the main transmission season model, except that *I* represents the number of infected bats from all the roosts that are mixing through swarming, and *N* the total population of the roosts that are mixing through swarming. An additional parameter, α, is also fitted to represent the relative between-roost transmission rate compared to the within-roost transmission rate during the swarming period. For the purposes of model fitting, we included the four roosts for which we have population size and serology data, and assumed potential swarming between them.

1. Hibernation (1 December – 30 April)

During hibernation it is assumed that disease progression is suspended because of cold temperatures and metabolic effects associated with torpor. It is further assumed that infectious bats die quickly and so the model describes overwinter mortality (for bats in the S, E and R classes) and disease-induced mortality (infectious bats).

$$\begin{matrix} \frac{dS_{j}}{dt}=-\mu_{pj}S_{j} \\ \frac{dE_{Rj}}{dt}=-\mu{}_{pj}E_{Rj} \\ \frac{dE_{Ij}}{dt}=-\mu_{pj}E_{\begin{matrix} Ij \\ \end{matrix}} \\ \frac{dR_{j}}{dt}=-\mu_{pj}R_{j} \\ \frac{dI_{j}}{dt}=-\nu I_{j} \end{matrix}$$

The above equations assume density dependent transmission (Figure S1). A frequency dependent transmission version of the equations was also fitted to the data (dividing the $\beta SI$ terms by *N*) (see Figure S2)

Summary of ABC approach

An ABC-Sequential Monte Carlo approach was used for the fitting of the model to the data, as described in Toni et al., 2009. This algorithm was initialised by sampling 1,000 values from the priors for the within and between roost rates of transmission, which were set as relatively uninformative: uniform in the range 0-1 for both density-dependent and frequency dependent transmission. To calculate the error metric between the model output and the observed data, the resulting differential equations for transmission were solved using the ode45 function in the software package Matlab, which is a Runge-Kutta method with variable time-step. For subsequent iterations, parameter values were sampled from the set of parameters for which the error between the model and the data was less than the error tolerance from the previous time step plus or minus a random perturbation; this perturbation was uniform with range +/- 20% of the parameter value, constrained to be within the range of the priors (greater proportions of the parameter value were also tried but did not affect model results). The model was run until no further reduction in error was possible.

# Results

### Correlates with immunity (statistical models)

Table S2: Additional results of univariate statistical modelling for weight, forearm length and year

| **Variable** | **N +ve / total** | **% +ve** | **Odds Ratio (95% CI)** | **p** |
| --- | --- | --- | --- | --- |
| **Weight** | 167/1839 |  | 1.23 (1.07-1.42) | 0.005 |
| **Forearm** | 167/1839 |  | 1.09 (0.95-1.26) | 0.202 |
| **Year** |  |  |  |  |
| 2004 | 0/16 | 0.0% | - |  |
| 2005 | 54/305 | 17.7% | 1 |  |
| 2006 | 29/284 | 10.2% | 0.53 (0.33-0.86) | 0.010 |
| 2007 | 15/189 | 7.9% | 0.40 (0.22-0.73 | 0.003 |
| 2008 | 17/291 | 5.8% | 0.29 (0.16-0.51 | <0.001 |
| 2009 | 10/317 | 3.2% | 0.15 (0.08-0.30 | <0.001 |
| 2010 | 19/253 | 7.5% | 0.38 (0.22-0.66 | 0.001 |
| 2011 | 11/107 | 10.3% | 0.53 (0.27-1.06 | 0.074 |
| 2012 | 12/78 | 15.4% | 0.85 (0.43-1.67 | 0.629 |

To account for possible differences between country, each univariate model had a random effect for country included (intercept, or intercept plus slope for continuous variables weight and forearm).

### Geographical variation

Analysis of the differences in seroprevalence between locations is complicated by different sampling strategies, low and different sample sizes from each location in Scottish and English locations. In multivariable analyses location is therefore included as a random variable. However, to attempt investigate differences between roosts, univariate model was also fitted to locations using English Roost A as a reference category (having largest sample numbers).

Table S3: Location

| **Covariate** | **N seropositive / N total** | **seroprevalence** | **Odds Ratio (95% CI)** | **p** |
| --- | --- | --- | --- | --- |
| **Location** |  |  |  |  |
| England A | 12/202 | 5.9% | 1 |  |
| England D | 21/135 | 15.6% | 2.81 (1.38-6.15) | 0.005 |
| East 1 | 6/20 | 30.0% | 3.35 (2.21-20.8) | 0.001 |
| East 3 | 9/34 | 26.5% | 3.55 (2.18-14.9) | 0.000 |
| East 4 | 4/20 | 20.0% | 2.17 (1.14-13.7) | 0.030 |
| East 6 | 15/85 | 17.7% | 2.97 (1.51-7.6) | 0.003 |
| North 2 | 15/87 | 17.2% | 2.90 (1.47-7.4) | 0.004 |
| Spare 13 | 4/20 | 20.0% | 2.17 (1.14-13.7) | 0.030 |
| *(other non-significant locations excluded from table)* |  |  |  |  |

### Multivariable models- assessment of fit:

Table S4. Multivariable model components

| **Term** | **Model A** | **Model B** | **Model C** |
| --- | --- | --- | --- |
| Weight | nd | nd | nd |
| Age | X | X | X |
| Sex | X | X | X |
| Reproductive status | X | nd | X |
| Month | X | nd | nd |
| Year | X | X | nd |
| Random effect for location | X | X | X |
| Number of parameters | 6 | 4 | 4 |
| AIC | 1071.9 | 1069.2 | 1107.1 |
| Hosmer-Lemeshow test | P=0.142 | P=0.132 | P=0.367 |
| c-statistic / ROC / AUC | 0.737 | 0.723 | 0.697 |

nd = parameter not included in model

Figure S1

Residual plots vs predicted values for model C:

|   Deviance residuals vs predicted probability of positivity |   Deviance vs sex |
| --- | --- |
|   Deviance residuals vs age category |   Deviance residuals vs reproductive category |
|   Deviance residuals vs month |  Deviance residuals vs year |

Residuals are not symmetrically distributed around zero due to the fact that the predicted seropositivity is generally low, so the animals that were seropositive (outcome=1) have a larger discrepancy between predicted (= a low probability) and observed (=1). The animals that were seronegative (observed=0), have a smaller discrepancy).

## ABC model results

**Figure S2. Posterior density plots**

**
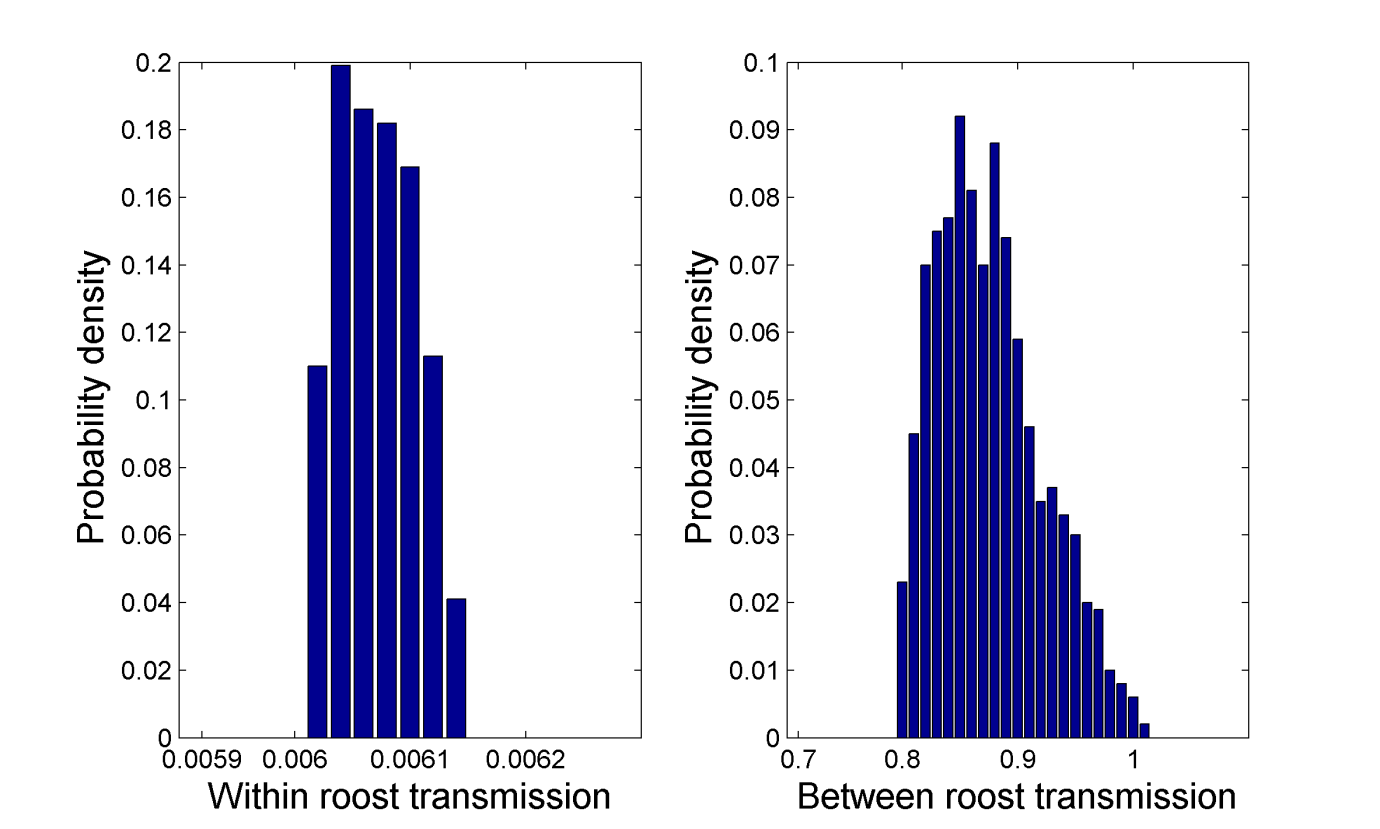
**

Figure S2. Probability density estimates of within (left hand plot) and between roost transmission (right hand plot), assuming density dependent transmission, estimated using approximate Bayesian computation applied to bat serology data.


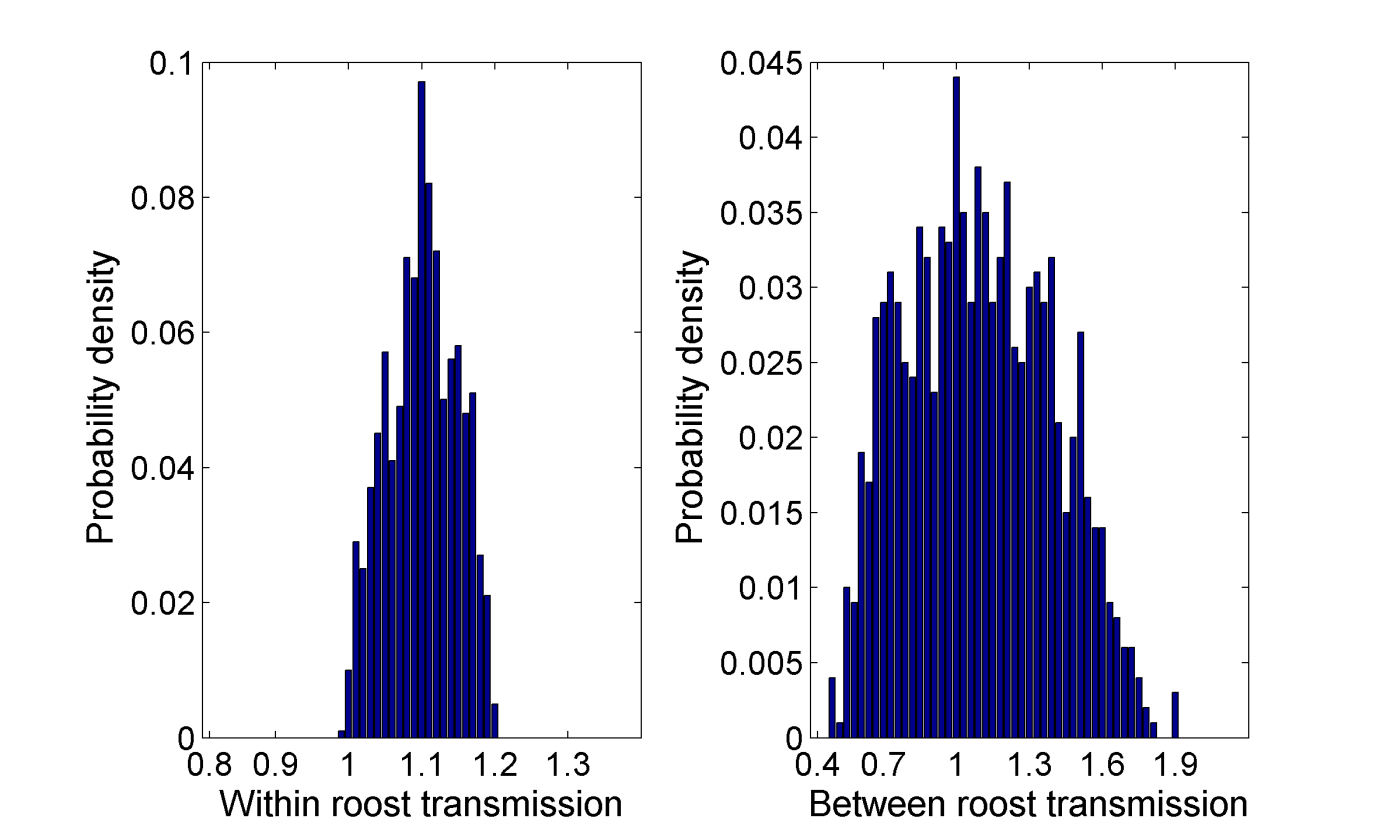


Figure S3. Probability density estimates of within (left hand plot) and between roost transmission (right hand plot), assuming frequency dependent transmission, estimated using approximate Bayesian computation applied to bat serology data.

**References**

1. Mitchell-Jones AJ, McLeish AP, Committee JNC. The Bat Worker's Manual: Joint Nature Conservation Committee (2004).

2. Racey PA, Swift SM, Mackie I. Recommended methods for bleeding small bats. *Acta Chiropterologica* **13**, 223-5 (2011).

3. Harris SL, Aegerter J, Brookes SM, McElhinney L, Jones G, Smith GC, et al. Targeted surveillance for European Bat lyssaviruses in English Bats (2003-2006). *Journal of wildlife diseases* **45**, 1030-41 (2009)*.*

4. Wakeley PR, Johnson N, McElhinney LM, Marston D, Sawyer J, Fooks AR. Development of a real-time, TaqMan reverse transcription-PCR assay for detection and differentiation of lyssavirus genotypes 1, 5, and 6. *Journal of clinical microbiology* **43**, 2786-92 (2005).

5. Webster WA. A tissue culture infection test in routine rabies diagnosis. Can J Vet Res. 1987;51(3):367-9. Epub 1987/07/01. PubMed PMID: 3651891; PubMed Central PMCID: PMC1255339.

6. White GC, Burnham KP. Program MARK: survival estimation from populations of marked animals. *Bird Study* **46**, 120-39.
